# King Edward VII.'s Hospital, Cardiff

**Published:** 1915-03-06

**Authors:** 


### King Edward VII.'s Hospital, Cardiff.

At the annual meeting of Governors last week, held under the presidency of the Lord Mayor of Cardiff (Alderman J. T. Richards), the speeches were of unusual interest. Major Ewen Maclean, in acknowledging the service of King Edward VII.'s Hospital to the 3rd Western General Hospital, said the general hospitals were under a deep debt to the voluntary hospitals, and the latter were among the brightest jewels in the Empire's crown. They were unique in their origin and equipment, for there was nothing approaching them in any other country. In five and a-half months 3,120 patients from overseas had been received, including 235 wounded Belgians. In addition they had had a number of home troops. In respect of the 3,120 men from the Expeditionary Force the mortality was only 0.2 per cent. Major Maclean paid a high tribute to Colonel Bruce Vaughan, who, as honorary advising architect, had transformed the schools into hospitals which had been adjudged by competent authorities to be not inferior to the equipped general hospitals of the county.

Principal Griffiths, in moving a vote of thanks to the honorary medical and surgical staff, said he had received that day a document which might lead them to believe that action regarding the Medical School would soon take place, and he trusted that those in a position to advise the Treasury would see to it that no niggardly grant came to the Medical School, but a grant that would indicate intelligent foresight and adequacy for so important a project. In referring to Sir William James Thomas's princely generosity for the purposes of the Medical School, Colonel Bruce Vaughan reminded the Governors that it was also Sir William's intention to present to the city and county a building in which the joint work of the public health departments might be carried out in the best and most scientific manner. It would be equal to the provision for these purposes at Manchester, and to that now being prepared in Liverpool. Colonel Vaughan referred to the need for a greater number of regular subscribers to the hospital, and appealed for funds to provide an additional operating theatre, a maternity ward for five or seven patients, wards for male and female tuberculous patients, and a chapel in which patients and staff might worship.

A healthy feature of the meeting was the large attendance of workmen's representatives, one of whom, who had joined the Colours since the outbreak of war, Driver J. Osmond, A.S.C., came, by the kind permission of his commanding officer, all the way from Deptford to attend the meeting.

